# Targeted RNA Degradation by RIBOTACs: A Novel Therapeutic Avenue for Ophthalmic Diseases

**DOI:** 10.3390/ijms27031493

**Published:** 2026-02-03

**Authors:** Dario Rusciano, Caterina Gagliano, Alessandro Avitabile, José Fernando Maya-Vetencourt

**Affiliations:** 1 Fidia Pharmaceuticals, Ophthalmology Research, Catania University, 95125 Catania, Italy; 2 Faculty of Medicine and Surgery, University of Enna “Kore”, 94100 Enna, Italy; 3 Mediterranean Foundation “G.B. Morgagni”, 95100 Catania, Italy; 4 Neurovisual Science Technology (NEST), 95121 Catania, Italy; 5 Department of Biology, Physiology Institute, University of Pisa, 56127 Pisa, Italy

**Keywords:** RIBOTACS, RNA degradation, RNA-based strategy, ribonuclease, ocular diseases, blindness

## Abstract

Ophthalmic diseases, including inherited retinal dystrophies, age-related macular degeneration (AMD), and glaucomatous neuropathies, are often driven by the expression of pathogenic proteins or dysfunctional non-coding RNAs that are currently considered ‘undruggable’ with conventional small-molecule therapeutics. The emerging strategy of Ribonuclease-Targeting Chimeras (RIBOTACs) offers a revolutionary approach to address this therapeutic gap. RIBOTACs are heterobifunctional small molecules designed to bind a specific target RNA with one moiety and recruit a latent endogenous ribonuclease, such as RNase L, with the other, thereby catalyzing the RNA’s degradation. This targeted degradation can potentially halt the production of mutant proteins, eliminate toxic gain-of-function RNAs, or modulate key regulatory pathways involved in angiogenesis, inflammation, and apoptosis—core processes in many blinding diseases. This review explores the immense potential of applying RIBOTAC technology to ophthalmology, discussing prospective targets such as mutant alleles in retinitis pigmentosa, VEGF transcripts in neovascular AMD, and inflammatory mediators in uveitis. We will also address the unique challenges and opportunities for RIBOTAC development in the eye, including delivery strategies to overcome ocular barriers, the need for high specificity to avoid off-target RNA degradation, and the optimization of pharmacokinetic properties for intraocular administration. With continued innovation, RIBOTACs are poised to evolve into a robust therapeutic platform, expanding the druggable genome and enabling precise, durable treatments for a range of currently intractable ophthalmic conditions.

## 1. Introduction

Over the past decade, the importance of RNA species in ocular biology and disease has become increasingly evident. MicroRNAs (miRNAs), long noncoding RNAs (lncRNAs), and messenger RNAs (mRNAs) contribute to key pathological processes in the eye—including inflammation, aberrant angiogenesis, fibrosis, and neurodegeneration—through highly interconnected regulatory networks. Numerous lncRNAs are differentially expressed in ocular tissues in conditions such as diabetic retinopathy, glaucoma, and age-related macular degeneration (AMD), suggesting their potential as therapeutic targets [1].

In diabetic retinopathy (DR), dysregulated noncoding RNAs influence vascular leakage, inflammation, and cell death [2,3] miRNAs modulate signaling pathways in endothelial cells, pericytes, and Müller glia, while lncRNAs contribute via epigenetic regulation and scaffolding of molecular complexes, fine-tuning pathological responses [4]. In AMD, integrative transcriptomic analyses have identified lncRNAs that are differentially expressed in the retinal pigment epithelium (RPE) and neural retina, implicating them in senescence, immune dysregulation, and complement activation [5].

Dysregulation of noncoding RNAs is also implicated in other proliferative retinopathies. In retinopathy of prematurity (ROP), miR-146a, miR-21, and miR-210 are downregulated in affected infants, with altered expression believed to influence angiogenic signaling [6]. Functional studies demonstrate that miR-126 suppresses VEGF expression and limits pathological neovascularization in the retina, providing mechanistic support for RNA dysregulation as a driver of disease phenotype [7]. Circular RNAs (circRNAs), acting as molecular “sponges” for miRNAs, add another regulatory layer: altered circRNA profiles have been documented in peripheral blood mononuclear cells of infants with ROP [8]. It has also been reported that circRNA RSU1 promotes retinal vascular dysfunction by regulating miR-345-3p/TAZ under diabetic conditions [9], illustrating the complexity of noncoding RNA networks in ocular disease.

Collectively, these findings underscore the central role of diverse RNA species in the molecular pathology of ophthalmic diseases, positioning them simultaneously as biomarkers and as promising therapeutic targets.

Despite this strong rationale, translating RNA-based therapies into the eye has been challenging. Small interfering RNAs (siRNAs), antisense oligonucleotides (ASOs), and miRNA mimics or inhibitors have shown promise in preclinical and early clinical studies, yet their impact is often limited by issues of delivery, stability, off-target effects, and immunogenicity. The eye’s compartmentalized anatomy facilitates local delivery. However, siRNA and ASO therapies still face significant challenges, including poor tissue penetration and transient pharmacological effects. ASOs have been especially explored in inherited retinal diseases: clinical trials for Leber congenital amaurosis associated with CEP290 mutations have demonstrated acceptable safety and early signs of efficacy [10,11]. However, the chemical nature of ASOs imposes significant drawbacks, including susceptibility to degradation, limited intracellular uptake, and potential immune activation. Improved chemistries, bio-conjugation strategies, and delivery platforms are needed to overcome these barriers [12,13].

The limitations of earlier RNA modalities are evident in the development of iCo-007, a second-generation ASO designed to downregulate multiple pro-angiogenic growth factors in DR. Although it exhibited an extended half-life, nuclease resistance, and a favorable safety profile, concerns persisted regarding dosing frequency, long-term durability, and the efficiency of retinal delivery [14]. Thus, despite significant progress in RNA therapeutics, a clear unmet need remains for approaches that are more potent, specific, and long-lasting.

In this landscape, Ribonuclease-Targeting Chimeras (RIBOTACs) have emerged as a highly compelling therapeutic modality (Table 1). RIBOTACs are bifunctional small molecules that bind a structured RNA target and simultaneously recruit an endogenous ribonuclease—typically RNase L—to catalyze its degradation [15]. By bringing RNase L into proximity with the target RNA, RIBOTACs trigger localized dimerization and activation of the enzyme, producing site-specific cleavage. This mechanism enables catalytic, rather than stoichiometric, target turnover: a single RIBOTAC molecule (differently from ASOs) can degrade multiple RNA copies. Moreover, because RIBOTACs rely on small-molecule recognition of RNA structural motifs rather than Watson–Crick base pairing, they can exploit unique three-dimensional RNA folds, offering the potential for enhanced specificity and reduced off-target interactions. Altogether, the RIBOTAC strategy directly addresses many of the longstanding challenges of RNA therapeutics and has the potential to deliver more durable and selective interventions for ocular disease [16,17]. This review discusses RIBOTACs’ mechanism of action, therapeutic potential, and challenges for ocular applications, while integrating recent advances in the field, thus providing a rationale for RIBOTACs’ use in ophthalmology. Importantly, RIBOTACs should not be viewed as competitors to established RNA-targeting platforms such as antisense oligonucleotides or RNA interference, which remain the most universal and clinically advanced approaches. Rather, RIBOTACs represent a mechanistically distinct, complementary strategy that may be advantageous in selected scenarios—particularly for structured RNAs that are poorly accessible to Watson–Crick base pairing or for applications where catalytic RNA degradation could offer enhanced potency or durability.

## 2. Mechanism of RIBOTACs

Over recent years, the mechanism of Ribonuclease-Targeting Chimeras (RIBOTACs) has been clarified, revealing a modular small-molecule architecture designed to achieve selective RNA degradation. RIBOTACs consist of three coordinated components: (i) an RNA-binding moiety that recognizes a specific structured RNA element, (ii) a recruiter module that engages an endogenous ribonuclease—most commonly RNase L—and (iii) a chemical linker optimized to permit simultaneous binding of both modules. The RNA-binding domain often derives from small molecules known to associate with defined RNA motifs; for example, *dovitinib* has been reprogrammed to bind the hairpin of pre-miR-21 and employed as a scaffold for RIBOTAC construction [18,19,20]. The recruiter module typically incorporates small-molecule RNase L activators. Early generations relied on 2′–5′ oligoadenylate analogs, whereas more recent designs use synthetic heterocycles identified through targeted screening [19,21,22]. Linker composition is critical: variations in length, rigidity, and permeability strongly influence ternary-complex formation and overall potency [20].

A central feature of RIBOTAC activity is RNase L biochemistry. RNase L is ordinarily a latent monomer, but upon recruitment it dimerizes and becomes catalytically active [23]. RIBOTACs promote this oligomerization by positioning an RNase L activator adjacent to the RNA-binding module. This proximity-driven assembly produces an RNase L–RNA–RIBOTAC ternary complex, enabling precise cleavage of the target RNA in cis [19,20,23]. Multiple experimental systems—such as saturation transfer difference (STD) NMR, recombinant RNase L assays, and cellular knockdowns—have been used to validate each mechanistic step from recruiter binding to RNA cleavage and degradation in living cells [23].

**Figure 1:**
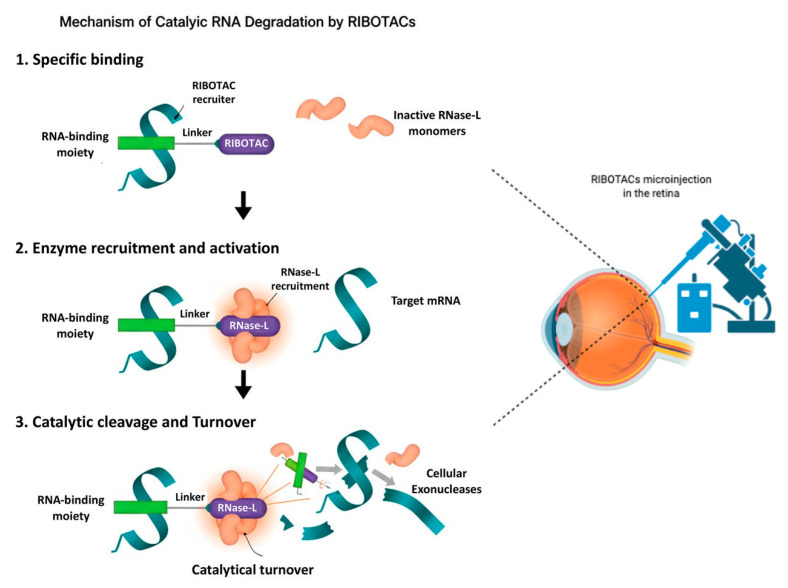
Mechanism of catalytic RNA degradation by RIBOTACs (left) and a schematic diagram of the eye and RIBOTAC delivery in the retina (right) highlighting the feasibility of application of this technology. RIBOTACs bind selectively to a target RNA through an RNA-binding moiety (Step 1), recruit and activate RNase L by inducing dimerization of inactive monomers (Step 2), and promote catalytic cleavage of bound RNA followed by exonucleolytic degradation and RIBOTAC turnover (Step 3). This may represent a novel way to target and degrade candidate RNAs involved in ocular diseases.

Selectivity arises from the ability of RIBOTACs to exploit the three-dimensional structure of RNA. Unlike oligonucleotide therapeutics, which rely on Watson–Crick complementarity, RIBOTACs recognize folded RNA motifs including hairpins, bulges, and internal loops [21,22]. Transcriptome-wide analyses show that only a subset of small-molecule-associated RNAs are efficiently cleaved, indicating that productive degradation requires both stable secondary structure and an accessible RNase L cleavage site adjacent to the binding motif [20]. Strategic placement of the recruiter module relative to natural RNase L cut sites is therefore essential (Figure 1). Degradation efficacy also varies with transcript abundance and with cell- or tissue-specific levels of RNase L, emphasizing the need to consider expression patterns and baseline enzymatic activity when selecting ocular RNA targets [20].

## 3. State of the Art: RIBOTACs in Other Diseases

Over the last few years, RIBOTACs have progressed rapidly from proof-of-concept molecules to therapeutically relevant agents across oncology, neurodegeneration, and other disease areas, demonstrating their versatility in targeting structured pathological RNAs.

### 3.1. Oncology Applications

One of the earliest and most extensively studied applications focuses on the oncogenic miRNA precursor pre-miR-21. *Dovitinib*, originally developed as a protein kinase inhibitor, was repurposed to bind the structured hairpin of pre-miR-21 and converted into a RIBOTAC by attaching an RNase L recruiter. This chimera selectively degraded pre-miR-21 in cells and exhibited markedly reduced activity against *dovitinib*’s native protein target, achieving a substantial shift in molecular specificity [18]. In mouse cancer models, systemic administration lowered miR-21 levels, restored tumor suppressor proteins such as PDCD4 and PTEN, and reduced metastatic progression [21].

To further refine precision, tumor-activated RIBOTACs (TaRiboTACs) have been developed to function only within the tumor microenvironment. A notable example is a dual-responsive TaRiboTAC engineered to degrade pre-miR-21 specifically in acidic and oxidative conditions. In this design, the RNase recruiter is masked with a phenylboronic acid group that is removed upon exposure to elevated hydrogen peroxide, thereby activating RNase L only in tumors [24]. Incorporation of an RGD peptide for tumor targeting and a fluorophore for imaging enabled both spatial selectivity and real-time tracking. In lung adenocarcinoma xenograft models, this molecule degraded pre-miR-21, enhanced radiosensitivity, and suppressed tumor growth [24].

RIBOTACs have also been extended to structured mRNAs and noncoding RNAs beyond miRNAs. Inducible RIBOTACs (iRIBOTACs), activated only in response to cell-specific triggers, have been developed to selectively degrade G-quadruplex (G4)-containing mRNAs, demonstrating the potential for precise spatial and temporal control in cancer therapy [25].

### 3.2. Neurodegenerative Diseases

RIBOTACs also show promise in neurodegenerative disorders in which aberrant RNA transcripts contribute directly to pathology. In Parkinson’s disease, pathological accumulation of α-synuclein is driven by elevated or dysregulated α-synuclein (SNCA) mRNA. Because the SNCA 5′ untranslated region contains a structured element, small molecules have been identified that bind this region and inhibit translation [26]. Building on this scaffold, researchers developed a RIBOTAC (“Syn-RiboTAC”) that selectively recruits RNase L to the SNCA transcript. In neuronal cultures, Syn-RiboTAC degraded SNCA mRNA, reduced SNCA protein levels, and restored expression of genes dysregulated in patient-derived neurons [26]. This illustrates how RIBOTACs can target transcripts underlying “undruggable” proteinopathies (Table 2).

Another important neurodegenerative target is the expanded G4C2 repeat in C9orf72, the most common genetic cause of amyotrophic lateral sclerosis (ALS) and frontotemporal dementia (FTD). Although peer-reviewed details remain limited, research reports and patent literature describe RIBOTACs designed to fold into the G-rich expanded RNA, recruit RNase L, and selectively degrade the pathogenic transcript [27]. Such strategies could simultaneously reduce nuclear RNA foci and the toxic dipeptide repeat proteins produced by repeat-associated non-AUG translation.

**Table 2:** Overview of key RIBOTAC molecules developed to date. For each, the table illustrates how RIBOTACs can degrade diverse RNA classes in different pathological contexts with high selectivity and efficacy.

RIBOTAC Name/Type	Target	Disease Model/Context	Key Findings/Results
TGP-21 RIBOTAC	pre-miR-21 (oncogenic miRNA)	Triple-negative breast cancer cells	Degraded pre-miR-21 with high potency, shifted selectivity away from the parent protein target, reduced mature miR-21, and was effective in mouse cancer models [18,19,21]
DEL-screened RNase L-recruiting RIBOTAC	pre-miR-21	Cancer cell lines	Identified a novel RNase L recruiter via DNA-encoded library (DEL) and combined it with an RNA-binding moiety to induce RNase L activation and pre-miR-21 cleavage [19]
TaRiboTAC (tumor-activated)	pre-miR-21	Tumor microenvironment, in vivo	A RIBOTAC activated by tumor-specific conditions (low pH, high H_2_O_2_) that selectively degrades pre-miR-21 in tumors, enhances radiosensitivity, and shows minimal systemic toxicity [16]
iRIBOTAC (inducible)	G-quadruplex (G4) RNAs	Cancer (in vitro/in vivo)	Designed for stimulus-dependent activation; upon triggering, it degrades G4-containing RNAs, induces apoptosis, and suppresses tumor growth in mice [25]
Syn-RiboTAC	SNCA mRNA (α-synuclein)	Parkinson’s disease (cell models, iPSC-derived neurons)	Selectively degrades SNCA mRNA, reduces α-synuclein protein, and rescues dysregulated gene expression in patient-derived neurons [26]
TERRA-RIBOTAC	lncRNA TERRA (G-quadruplex)	ALT cancer cell lines	Targets the G-quadruplex structure of TERRA, recruits RNase L, degrades TERRA, disrupts telomere maintenance, and reduces clonogenic survival in cancer cells [28]

### 3.3. Other RNA Structures

Beyond mRNAs and miRNAs, RIBOTACs are being engineered against structurally complex long noncoding RNAs. A prime cancer target is the lncRNA TERRA, which forms G-quadruplex structures crucial for telomere maintenance in tumors that rely on the Alternative Lengthening of Telomeres (ALT) pathway. By designing RIBOTACs that bind TERRA’s G-quadruplexes and recruit RNase L, researchers can induce its degradation. This strategy directly impairs telomere maintenance, leading to significantly reduced clonogenic survival in ALT cancer models [28].

In parallel with therapeutic development, new tools have been introduced to visualize and quantify RNase L activity in real time. Fluorescent RNase L probes that emit a signal upon binding allow live-cell imaging of enzyme activation, trafficking, and localization [29]. These tools also facilitate high-throughput screening of recruiter modules and provide a platform for optimizing RIBOTAC chemistry and mechanism in diverse cellular systems.

## 4. Potential Ophthalmic Applications of RIBOTACs

The demonstrated utility of RIBOTACs in oncology and neurodegeneration highlights a strong opportunity to translate this platform into ophthalmology. The eye—particularly the retina—is increasingly recognized as a site of profound RNA dysregulation, with mounting evidence implicating RNA mis-splicing, aberrant noncoding RNA activity, and pathological RNA–protein interactions in diseases such as age-related macular degeneration (AMD) [30]. This expanding understanding of RNA-centric mechanisms provides a compelling rationale for applying targeted RNA degradation strategies such as RIBOTACs.

### 4.1. Candidate RNA Targets in Eye Diseases

A broad range of regulatory RNAs contribute to ocular pathology, making them strong candidates for targeted degradation. Among microRNAs, miR-21 is one of the most extensively implicated in diabetic retinopathy. Elevated miR-21 correlates with disease severity in patient plasma [31,32], and functionally it suppresses PPARα, promoting vascular dysfunction; inhibition of miR-21 ameliorates microvascular damage in experimental models [33]. Another key regulator, miR-146a, plays an anti-inflammatory role in retinal endothelial cells under hyperglycemic stress, reducing IL-6, STAT3 phosphorylation, and VEGF expression [34]. Multiple additional miRNAs—including miR-200b, miR-126, miR-195, and miR-29b—are involved in angiogenesis and regulated cell death pathways in DR [32], while miR-34a, miR-27a, miR-23a, and miR-155 have been associated with degeneration and immune dysregulation in AMD [35]. In inherited conditions, miR-184 is strongly implicated in keratoconus and cataract, reflecting its essential role in corneal and aqueous humor homeostasis [36,37,38,39].

Long noncoding RNAs also play prominent roles in retinal vascular disease. Among them, MALAT1 has emerged as a particularly relevant therapeutic target. MALAT1 contributes to oxidative stress, angiogenesis, and inflammation in DR [40,41], and its knockdown reduces pericyte loss, capillary degeneration, vascular leakage, and inflammatory cytokine expression [42]. Mechanistically, MALAT1 functions as a competing endogenous RNA—binding miR-124 to promote MCP-1-mediated inflammation [43] and sequestering miR-125b to enhance VE-cadherin expression and angiogenesis [44]. Under high-glucose stress, MALAT1 is transcriptionally upregulated via Sp1, suppressing Nrf2 signaling and increasing oxidative damage [40]. It also modulates the miR-320a/HIF-1α axis and influences endothelial–mesenchymal transition via miR-205-5p/VEGF-A [42,45]. Alongside MALAT1, the lncRNA XIST is another candidate target given its documented expression and regulatory activity in retinal cells [46,47].

Together, these RNAs represent central nodes in the pathogenic networks driving retinal inflammation, oxidative stress, and neovascularization. Strategic degradation of key transcripts such as miR-21 or MALAT1 could modulate multiple disease pathways simultaneously. However, optimal therapeutic benefit will depend on aligning target selection with disease stage and cell-type-specific expression—for example, enhancing miR-146a activity early in DR to suppress inflammation versus targeting MALAT1 in later proliferative stages to curb neovascular growth.

### 4.2. Design Strategies for Ocular RIBOTACs

Adapting RIBOTACs for ophthalmic use requires careful attention to molecular design and delivery. The RNA-binding module must recognize native folded structures—such as miRNA hairpins or lncRNA loop domains—within retinal cells. This domain must then be linked to an RNase L–recruiting moiety via a linker optimized for stability, cell permeability, and productive ternary-complex formation. Because RNase L expression and activation thresholds differ across retinal endothelial cells, Müller cells, and RPE, recruiter potency may need to be tuned to the specific cellular environment.

Conditional activation strategies, such as masked recruiters that respond to oxidative stress or inflammatory signals, may enhance local specificity by initiating RNase L activation only within diseased tissue. This approach mirrors successful tumor-activated RIBOTAC designs and could reduce off-target degradation in healthy retinal regions [24].

Delivery remains a central challenge due to the blood–retinal barrier and the viscoelastic vitreous. Non-viral nanocarriers—including solid lipid nanoparticles (SLNs), liposomes, and hydrogels—can increase stability, enable sustained release, and improve posterior-segment penetration. Lipid nanoparticles (LNPs), in particular, have demonstrated efficient retinal delivery after intravitreal injection, supporting mRNA expression in mouse and human retina without acute inflammation [48]. Reviews of ocular nanocarrier systems highlight SLNs and nanostructured lipid carriers (NLCs) as promising vehicles due to their biocompatibility, sustained release properties, and favorable biodistribution [49,50]. Additional control over specificity may come from “activation-upon-disease” paradigms, in which linkers or recruiters respond to pathological microenvironment cues such as oxidative stress or inflammatory mediators [51].

Of note, targeted protein degradation strategies have already been explored in ophthalmology. The proteolysis-targeting chimera (PROTAC), known as dBET6, actually inhibits the Bromodomain and ExtraTerminal domain (BET) proteins while reducing light-induced retinal degeneration and suppressing the cGAS–STING pathway of inflammation in microglia and macrophages [52], illustrating the feasibility of deploying heterobifunctional small-molecule degraders in retinal disease and providing an encouraging precedent for RIBOTAC-based therapies.

## 5. Challenges and Limitations

Although RIBOTACs offer a powerful and elegant strategy for targeted RNA degradation, they currently lag behind RNAi and ASO therapeutics in clinical maturity, delivery optimization, and breadth of applicable targets. Their translation to ocular therapy, furthermore, poses substantial scientific, safety, and regulatory challenges. Multiple layers of complexity must be addressed to ensure that RIBOTACs can be deployed safely and effectively within the highly specialized and immune-sensitive environment of the eye.

### 5.1. Safety and Toxicity

A central concern is the risk of excessive or dysregulated RNase L activation. Strong or prolonged recruitment of RNase L may cause cleavage of unintended bystander RNAs, generating self-RNA fragments that activate pattern-recognition receptors such as RIG-I and DHX15, thereby amplifying interferon signaling and inflammasome pathways [53,54]. Canonical studies also demonstrate that RNase L–derived self-RNAs can stimulate the RIG-I/MDA5 axis, potentiating antiviral and inflammatory responses [55]. Overactivation of RNase L further risks inducing ribotoxic stress: evidence indicates that sustained cleavage activity can trigger ZAKα-mediated MAPK activation (JNK/p38), producing inflammatory and cytotoxic outcomes [56].

These responses constitute major safety liabilities for ocular therapy, where inflammation—even if transient—can cause irreversible structural or functional damage. Because RIBOTACs function by enforcing proximity between RNase L and the target RNA, minimizing off-target cleavage and limiting excessive activation will be essential. Balancing recruitment strength, tuning linker geometry, and using conditional activation mechanisms will likely be necessary to maintain therapeutic specificity.

A related issue is that RNase L is an integral component of the innate antiviral defense system. Its activation by a RIBOTAC can mimic viral RNA cleavage and generate potent immune stimuli. RNase L–derived RNA fragments promote antiviral stress granule formation, boost interferon production via RIG-I and PKR, and induce pro-inflammatory transcriptional programs [53,54]. In addition, cleavage products can activate the NLRP3 inflammasome, leading to IL-1β release [57]. These findings underscore the need for strict control of RNase L activation when deploying RIBOTACs in the retina.

Off-target RNA cleavage represents a central safety concern for any ophthalmic RIBOTAC therapy. Although RNase L is not a globally destructive nuclease, transcriptome-wide analyses in mammalian cells have shown that it cleaves a defined but not fully exclusive subset of cellular RNAs [20,58]. This means that even when a RIBOTAC is designed to recruit RNase L to a specific pathogenic transcript, bystander RNAs may still be degraded, particularly those that naturally contain RNase-L–preferred UN^N motifs or become locally accessible during stress. For this reason, comprehensive and unbiased off-target profiling is essential. Techniques such as RNA-seq, PARE/GMUCT, and 5′-end–mapping approaches can precisely identify all RNA fragments generated after RIBOTAC treatment, allowing investigators to distinguish intended target cuts from unintended cleavage products [59,60].

### 5.2. Efficiency of Degradation

Binding to a target RNA does not guarantee efficient degradation. Effective cleavage requires that the recruiter module be positioned at a conformationally appropriate distance from accessible RNase L cleavage sites. Transcriptome-wide studies confirm that only a subset of small-molecule–bound RNAs are efficiently degraded; RNAs with stable local structures around the binding pocket and proximal RNase L cut sites are more susceptible to cleavage [20].

Design, therefore, depends on careful structural characterization of the target RNA using SHAPE, in-line probing, or biochemical footprinting to identify both the structural binding motif and adjacent cleavage sites [23].

Kinetic factors further influence degradation efficiency. The catalytic cycle relies on a “bind–cleave–leave” mechanism: RNase L must cleave the RNA efficiently, and the RIBOTAC must dissociate rapidly enough to recycle to new RNA molecules. Overly strong binding can decrease turnover, while weak binding reduces on-target engagement. Modeling of enzyme-recruiting oligonucleotides shows that excessively tight binding reduces catalytic recycling [61]. Experimental systems demonstrate that optimal designs allow efficient dissociation and repeated cycles of cleavage [62]. Finally, potent RNase L activators remain rare [63], highlighting the difficulty of balancing activation strength with catalytic turnover.

### 5.3. RNase L Expression and Tissue Specificity

RNase L is a ubiquitously expressed endoribonuclease that functions as a central effector of the interferon-stimulated antiviral response. Under basal conditions, RNase L exists predominantly in an inactive monomeric state, and its catalytic activity is tightly regulated to prevent unintended RNA degradation [52,55]. Both expression levels and activation thresholds appear to vary across tissues and cell types, reflecting differences in innate immune tone and stress responsiveness [55,57].

In ocular tissues, direct quantitative mapping of RNase L expression remains limited. However, transcriptomic datasets and interferon-response studies indicate that RNase L is broadly expressed in multiple ocular cell populations, including retinal pigment epithelium and immune-associated cells, consistent with its widespread tissue distribution [52,55]. Importantly, RNase L expression and responsiveness are inducible and may be enhanced under inflammatory or degenerative conditions, including those characterized by heightened innate immune signaling, such as diabetic retinopathy, uveitis, or retinal injury [52,53,54].

To date, RNase L has not been identified as a causative factor in ophthalmic diseases, nor has its dysregulation alone been linked to retinal pathology. Instead, RNase L should be viewed as a conditionally activatable enzymatic effector whose activity is normally constrained and context-dependent [55,57]. This suggests that RNase L recruitment by RIBOTACs represents a pharmacologically imposed function rather than amplification of an endogenous disease mechanism.

Nevertheless, variability in RNase L abundance, inducibility, and downstream immune signaling across retinal cell populations may strongly influence both efficacy and safety. Previous studies demonstrate that RNase L-mediated RNA cleavage can generate self-RNA fragments capable of activating RIG-I–dependent innate immune pathways, underscoring the importance of controlled and localized activation [53,54]. Consequently, systematic characterization of RNase L expression and activation capacity in healthy versus diseased ocular tissues—using approaches such as single-cell RNA sequencing, spatial transcriptomics, and activity-based probes—will be essential for the rational development of ophthalmic RIBOTACs.

### 5.4. Pharmacokinetics and Pharmacodynamics in the Eye

Achieving therapeutic concentrations of RIBOTACs in the eye presents formidable pharmacokinetic (PK) and pharmacodynamic (PD) challenges. Small molecules may degrade chemically or enzymatically in aqueous and vitreous humor, as shown in ex vivo vitreous studies of protein and small-molecule therapeutics [64]. Intravitreal models further indicate that dissolution, instability, and clearance can markedly affect vitreous residence time [65].

The vitreous itself—a hydrogel composed of collagen fibrils and polyanionic glycosaminoglycans—imposes selective permeability that varies with molecular size, charge, and hydrophobicity [66]. PK models demonstrate that the balance between anterior and posterior elimination pathways, diffusion coefficients, and molecular dimensions governs intraocular half-life [67]. Both modeling and experimental work show that lipophilicity and molecular size strongly influence clearance, with small lipophilic molecules exhibiting rapid posterior elimination [67,68]. Preclinical studies confirm that small molecules typically diffuse rapidly unless stabilized or encapsulated within a delivery system [69].

Optimizing dose and dosing frequency will therefore be essential. Depending on therapeutic index and RNase L activation kinetics, RIBOTACs may require more frequent low-dose intravitreal injections or sustained-release delivery strategies. Controlled-release modalities—including PEGylated prodrug conjugates, biodegradable implants, and nanoparticle depots—can extend intraocular residence time. For example, a 4-arm PEG–small-molecule conjugate achieved a vitreous half-life of ~7 days in rabbits, with human simulations predicting monthly dosing intervals [70]. PEG-PCL-TMC nanocarriers provide retention for 4–30+ days in rabbits [71], and multiple preclinical systems demonstrate months-long release of small molecules from ocular implants [72]. A recent review highlights the potential of biodegradable nanocarriers to reduce injection burden and improve PK profiles [73].

### 5.5. Translational and Regulatory Hurdles

Translational development will depend heavily on selecting appropriate disease models. Many rodent models of diabetic retinopathy exhibit delayed or incomplete phenotypes, limiting the evaluation of chronic vascular and neuroinflammatory changes [74]. Species differences in retinal architecture and RNase L regulation may also affect translational extrapolation, increasing the importance of large-animal or primate models [75].

Manufacturing RIBOTACs introduces additional complexity. These heterobifunctional molecules integrate an RNA-binding module, a recruiter, and a linker; scaling production requires precise control of stereochemistry, linker composition, and impurity profile. Regulatory agencies will expect rigorous characterization of batch reproducibility, stability, and chemical identity.

As a novel therapeutic modality, RIBOTACs will likely undergo heightened regulatory scrutiny. Given their catalytic mechanism and potential immune activation, safety packages must include comprehensive tissue-specific activation data, immunogenicity assessments, and unbiased off-target profiling. Regulatory frameworks for oligonucleotide therapeutics provide partial guidance: clinical pharmacology programs must evaluate immunogenic risk, characterize biodistribution, and monitor unintended immune activation [76]. Immunogenicity evaluation for nucleic acid conjugates often requires validated anti-drug assays and risk-based strategies tailored to molecular design [77].

## 6. Future Directions and Perspectives

Translating RIBOTACs into ophthalmic therapeutics will require a structured preclinical roadmap (Table 3). Rigorous target validation is the first essential step: once candidate miRNAs or lncRNAs are identified, their susceptibility to RIBOTAC-mediated RNase L recruitment must be confirmed in vitro using disease-relevant ocular cell types such as retinal pigment epithelium (RPE), Müller glia, and retinal endothelial cells. Subsequent in vivo studies in appropriate disease models—such as diabetic retinopathy or AMD—will be crucial. These experiments must be accompanied by detailed mapping of RNase L expression and activation potential in retinal tissues to ensure that the enzyme is present and capable of mediating catalytic RNA degradation.

RIBOTACs also hold promise for combination therapy. A RIBOTAC targeting a pro-angiogenic miRNA could complement anti-VEGF treatments, potentially reducing injection frequency or dose. Likewise, combination with gene therapies represents a synergistic opportunity: for example, in a porcine neovascular AMD model, AAV-delivered shRNA targeting VEGFA produced robust anti-angiogenic effects [78]. Pairing the catalytic action of a RIBOTAC with the durability of AAV-mediated gene suppression could provide multi-layered therapeutic benefit.

The emerging relevance of personalized medicine further strengthens the case for precision RNA-targeting strategies. Noncoding RNA signatures vary substantially between individuals—circulating miRNA profiles differ among patients with diabetic retinopathy [79] and AMD [80], while lncRNA expression patterns, such as LINC00276 in AMD, show patient-specific variability [5]. RIBOTACs could therefore be tailored to the dominant pathological RNA species in each patient, enhancing both therapeutic efficacy and safety.

Technological innovation will play a key role in the next generation of ocular RIBOTACs. Conditional or prodrug RIBOTACs—such as “caged” designs that remain inert until a physiological trigger removes a protective group—have recently been demonstrated using a bioorthogonal RNase L recruiter system [51]. Stimulus-responsive strategies, including light-activated RIBOTACs, could enable precise spatial or temporal control of RNase L activation in diseased retinal regions. Advances in live-cell RNA imaging are similarly transformative: split-aptamer and split-protein fluorescent systems now permit visualization of RNA interactions in real time [81], providing a platform for monitoring RIBOTAC engagement, activation, and activity in living tissue.

In parallel with small-molecule RNA degraders, CRISPR–Cas13-based systems have emerged as a highly programmable platform for RNA targeting, including messenger RNAs and long noncoding RNAs. Cas13 enzymes enable sequence-specific RNA cleavage or editing guided by customizable CRISPR RNAs, offering broad versatility and strong knockdown efficiency across diverse RNA species [82,83]. In the context of ophthalmology, recent studies have demonstrated the feasibility of delivering compact Cas13 variants to the retina using viral vectors, achieving efficient RNA modulation with functional and structural preservation in preclinical models [84]. These findings support the potential of Cas13 systems as a gene-therapy-like approach for selected inherited or acquired ocular disorders.

However, important challenges remain, particularly for chronic retinal diseases. Cas13-based strategies typically require sustained expression of a bacterial nuclease, raising concerns related to immunogenicity, long-term safety, and limited reversibility. In addition, certain Cas13 orthologs have been shown to induce collateral RNA cleavage in human cells, with temporal dynamics that may complicate precise control of RNA degradation and increase the risk of unintended transcriptome perturbation [85]. Consequently, while CRISPR–Cas13 represents a powerful and versatile RNA-targeting modality, its translational profile differs fundamentally from that of RIBOTACs. Cas13 approaches may be best suited for durable, gene-therapy–style interventions, whereas RIBOTACs offer a pharmacologically tunable and potentially reversible alternative that exploits endogenous RNA decay machinery rather than permanent nuclease expression.

In summary, the integration of rigorous target validation, optimized preclinical modeling, rational molecular design, and next-generation activation or imaging technologies provides a clear roadmap for translating RIBOTACs into ophthalmic therapy. If these components can be successfully aligned, RIBOTACs have the potential to inaugurate a new class of catalytic RNA-degrading therapeutics for retinal disease.

## 7. Conclusions

RIBOTACs represent a compelling new strategy for selectively targeting pathogenic RNAs in ocular disease. By recruiting the endogenous endoribonuclease RNase L, they enable catalytic degradation of disease-driving transcripts, potentially achieving greater potency and durability than traditional antisense or small-molecule approaches. Their bifunctional architecture—combining structure-specific RNA binding with localized enzyme recruitment—provides the basis for high specificity in selected, structurally defined RNA targets, complementing the broader versatility of sequence-based RNAi and antisense approaches.

However, translating RIBOTACs into ophthalmic therapeutics will require overcoming substantial barriers, including delivery challenges, pharmacokinetic constraints, off-target risks, and the need for tight control of RNase L activity. Progress will depend on rigorous target validation, careful molecular design, innovative activation strategies, and comprehensive preclinical testing. Despite these hurdles, the potential impact of ocular RIBOTACs is significant: the ability to catalytically degrade pathologic RNAs positions them as a fundamentally new modality for treating retinal diseases driven by complex RNA dysregulation.

Over the next decade, translation is likely to follow a staged developmental roadmap. In the near term, optimized RIBOTAC candidates directed against validated retinal miRNAs or lncRNAs will undergo systematic evaluation in rodent and large-animal models to define pharmacokinetics, biodistribution, and safety. As formulation approaches mature, sustained-release or conditionally activated RIBOTACs may enter early-phase clinical trials for diabetic retinopathy or neovascular AMD—potentially in combination with anti-VEGF agents to reduce treatment burden. Personalized applications may also emerge, leveraging patient-specific RNA signatures in vitreous or blood to guide target selection and stratify therapy.

Concurrently, advances in chemical biology—such as inducible or “caged” RIBOTACs and light-activated recruit-and-degrade systems—may enable precise spatial or temporal control in retinal tissue, improving safety and minimizing off-target effects. The development of noninvasive imaging probes capable of tracking RNase L activation in vivo could provide real-time pharmacodynamic biomarkers, accelerating dose optimization and clinical monitoring. Last, different experimental strategies such as the use of ocular implants or gene editing vectors, in combination with the administration of RIBOTACs in the retina, may also be of clinical relevance in the treatment of eye disorders. Another safety consideration is that novel chemical- and stimulus-responsive approaches, as well as the optimization of RIBOTAC design, might be important to mitigate the cleavage of unintended RNAs, thus minimizing the potential undesired impact of this strategy.

If these scientific and technological developments converge, RIBOTACs could inaugurate a transformative class of RNA-degrading therapeutics in ophthalmology, offering disease-modifying treatments for conditions that currently rely on symptomatic management.

## Figures and Tables

**Table 1:** Comparison of antisense oligonucleotides (ASOs) and RIBOTACs. The table highlights mechanistic and developmental differences between the two platforms; these approaches should be considered complementary rather than mutually exclusive.

Feature	Antisense Oligonucleotides (ASOs)	RIBOTACs
Mechanism	Base pairing; recruits RNase H or steric block	Bifunctional chimera; recruits cellular ribonuclease (e.g., RNase L)
Specificity	High (sequence-based)	Very high/theoretically higher (dual-recognition)
Potency	Stoichiometric (1:1 ratio)	Catalytic (highly potent, amplifies effect)
Delivery	Mature (chemical modifications, approved drugs)	Nascent/challenging (pre-clinical stage)
Clinical Status	Multiple FDA-approved drugs	Research-stage, no approved drugs
Major Advantage	Proven platform, predictable design	High specificity and potency, can target undruggable RNAs
Major Disadvantage	Off-target effects, delivery limitations	Difficulty finding RNA-binding molecules, unproven delivery

**Table 3:** Key risks and mitigation strategies for ocular RIBOTAC development. This table summarizes potential safety, efficacy, pharmacokinetic, and translational risks associated with using RIBOTACs in the eye—such as uncontrolled RNase L activation, off-target RNA cleavage, poor ocular retention, and manufacturing hurdles—along with proposed strategies to minimize them, including conditional activation, structural optimization, controlled-release delivery, and rigorous preclinical evaluation.

Risk/Challenge	Potential Consequences	Mitigation Strategy
Safety/Toxicity	Excessive or uncontrolled RNase L activation → unintended degradation of non-target RNAs, cytotoxicity, and inflammation	- Titrate recruiter strength; use weak-to-intermediate-affinity recruiters—perform transcriptome-wide RNA-seq after treatment to identify off-target cleavage—engineer “conditional” activation (e.g., masked recruiter that only activates under oxidative stress or inflammation)—test immune activation (e.g., interferon response) in ocular cell lines or animal models
Immune Responses	RNase L activation mimicking antiviral response → cytokine/IFN induction, immune damage	- Monitor cytokine/interferon-stimulated gene induction—use inducible RIBOTACs that are only activated in diseased tissue—evaluate local (intraocular) immune effects in preclinical models
Off-Target Cleavage	Unintended cleavage of unrelated RNAs → disruption of important cellular RNAs	- Map RNase L cleavage preferences around the target (e.g., via structural mapping)—use high-resolution profiling (RNA-seq, cleavage assays)—optimize RIBOTAC design (linker length, binding module) to favor on-target cleavage
Variable Degradation Efficiency	Some target RNAs may bind but not be efficiently cleaved	- Use structural probing (e.g., SHAPE, in-line probing) to identify favorable binding/cleavage sites—optimize linker design and recruiter geometry to maximize proximity to cleavage sites—measure RIBOTAC turnover and recycling kinetics in cells
Kinetics/Recycling	Limited “recycling” of RIBOTAC or slow cleavage → reduced potency	- Quantify binding/dissociation kinetics—modify chemical moieties to improve dissociation rate or recurrence—design RIBOTACs with favorable off-rates and then test long-term cellular exposure
Stability in Intraocular Fluids	Degradation, aggregation, or inactivation of RIBOTAC in vitreous or aqueous humor	- Evaluate chemical stability in vitro in simulated vitreous/aqueous environments—use protective formulations (e.g., encapsulation in nanoparticles)—stabilize molecule via chemical modification (PEGylation, prodrugs)
Retention/Clearance in the Eye	Rapid clearance → insufficient exposure or accumulation → toxicity	- Measure pharmacokinetics (PK) in ocular compartments (vitreous, retina)—use sustained-release delivery systems (e.g., biodegradable implants, injectable depots)—optimize dosing frequency based on PK/PD data
Dose Optimization	Too high → toxicity; too low → inefficacy	- Carefully titrate dose in preclinical models (both normal and disease)—conduct dose–response studies—use controlled-release delivery to smooth out exposure
Translational/Animal Model Relevance	Animal models may not reflect human RNase L expression or activation	- Quantify RNase L expression and activity in animal retina/RPE—use humanized or disease-relevant models—validate RIBOTAC activity in ex vivo human ocular tissues if possible
Manufacturing/Scale-Up	Difficulty in synthesizing complex bifunctional molecules at scale	- Develop robust synthetic routes, use modular synthesis—characterize batch-to-batch consistency (purity, activity)—work with CMC (chemistry, manufacturing, controls) experts early
Regulatory Hurdles	Novel modality → extensive safety, off-target, immunogenicity data required	- Design preclinical safety studies that include transcriptome profiling, cytokine assays, immunogenicity—provide data on tissue-specific activation—engage regulatory agencies early to define acceptable safety margins

## Data Availability

No new data were created/analyzed in this study. Data sharing is not applicable to this manuscript.
